# Supplementary material for: Comparison of Various Solvent Extracts and Major Bioactive Components from Unsalt-Fried and Salt-Fried Rhizomes of Anemarrhena asphodeloides for Antioxidant, Anti-α-Glucosidase, and Anti-Acetylcholinesterase Activities
Source: Antioxidants (Basel). 2022 Feb 14;11(2):385. doi: 10.3390/antiox11020385 (PMC8868586; doi:10.3390/antiox11020385)
Supplement: Supplementary file 1 [file antioxidants-11-00385-s001.zip › antioxidants-1595439-supplementary.pdf]

Supplementary data

Comparison of Various Solvent Extracts and Major Bioactive Components from Unsalt-fried and Salt-fried Rhizomes of *Anemarrhena asphodeloides* for Antioxidant, Anti-α-Glucosidase, and Anti-Acetylcholinesterase Activities

Yi-Cheng Chu [1], Chang-Syun Yang [2], Ming-Jen Cheng [3], Shu-Ling Fu [1,*] and Jih-Jung Chen [1,2,4,*]

[1] Institute of Traditional Medicine, School of Medicine, National Yang Ming Chiao Tung University, Taipei 112, Taiwan; chuyc.md07@nycu.edu.tw (Y.-C.C.)

[2] Department of Pharmacy, School of Pharmaceutical Sciences, National Yang Ming Chiao Tung University, Taipei 112, Taiwan; tim0619@nycu.edu.tw (C.-S.Y.)

[3] Bioresource Collection and Research Center (BCRC), Food Industry Research and Development Institute (FIRDI), Hsinchu 300, Taiwan; cmj@firdi.org.tw (M.-J.C.)

[4] Department of Medical Research, China Medical University Hospital, China Medical University, Taichung 404, Taiwan

* Correspondence: jjungchen@nycu.edu.tw (J.-J.C.); slfu@nycu.edu.tw (S.-L.F.); Tel.: +886-2-2826-7195 (J.-J.C.); +886-2-2826-7177 (S.-L.F.); Fax: +886-2-2823-2940 (J.-J.C.); +886-2-2822-5044 (S.-L.F.)

Contents

Table S1. Retention time, LODs, LOQs, and regression analysis for three components of *Anemarrhena asphodeloides* (AA) in reverse-phase HPLC.. S3

Figure S1. Reverse-phase HPLC chromatogram of isolated pure compounds........................ S3

Figure S2. Reverse-phase HPLC chromatogram of methanol extract in AA............................ S3

Figure S3. Reverse-phase HPLC chromatogram of ethanol extract in AA................................S4

Figure S4. Reverse-phase HPLC chromatogram of acetone extract in AA................................S4

Figure S5. Reverse-phase HPLC chromatogram of ethyl acetate extract in AA....................... S4

Figure S6. Reverse-phase HPLC chromatogram of CH_2Cl_2 extract in AA................................ S5

Figure S7. Reverse-phase HPLC chromatogram of *n*-hexane extract in AA.............................S5

Figure S8. Reverse-phase HPLC chromatogram of water extract in AA..................................S5

Figure S9. Reverse-phase HPLC chromatogram of methanol extract in salt-fried AA.......... S6

Figure S10. Reverse-phase HPLC chromatogram of ethanol extract in salt-fried AA............ S6

Figure S11. Reverse-phase HPLC chromatogram of acetone extract in salt-fried AA............ S6

Figure S12. Reverse-phase HPLC chromatogram of ethyl acetate extract in salt-fried AA.... S7

Figure S13. Reverse-phase HPLC chromatogram of CH_2Cl_2 extract in salt-fried AA............. S7

Figure S14. Reverse-phase HPLC chromatogram of *n*-hexane extract in salt-fried AA…........S7

Figure S15. Reverse-phase HPLC chromatogram of *n*-hexane extract in salt-fried AA...........S8

Table S1. Retention time, LODs, LOQs, and regression analysis for four components of *Anemarrhena asphodeloides* (AA) in reverse-phase HPLC.

Compounds	T_m (min) [a]	Regression equation	Correlation coefficient	LOD (μg/mL) [a]	LOQ (μg/mL) [a]
Neomangiferin	28.23	y = 32979x + 5.8829	0.9999	0.18	0.56
Timosaponin AIII	31.37	y = 1.3673x – 1.6725	0.9999	0.20	0.48
Isomangiferin	33.25	y = 4.1050x – 2.0690	0.9998	0.10	0.43
Mangiferin	59.82	y = 3.7650x + 5.2980	0.9997	0.16	0.50

[a] T_m: Retention time; LOD: Limit of detection; LOQ: Limit of quantification

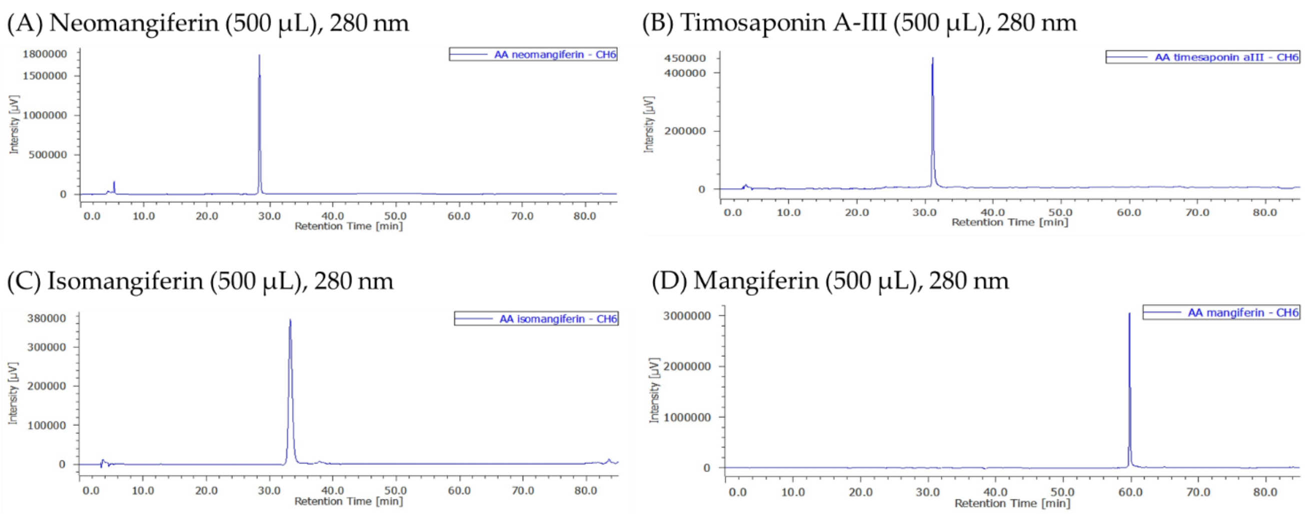

Figure S1. Reverse-phase HPLC chromatogram of isolated pure compounds (A to D).

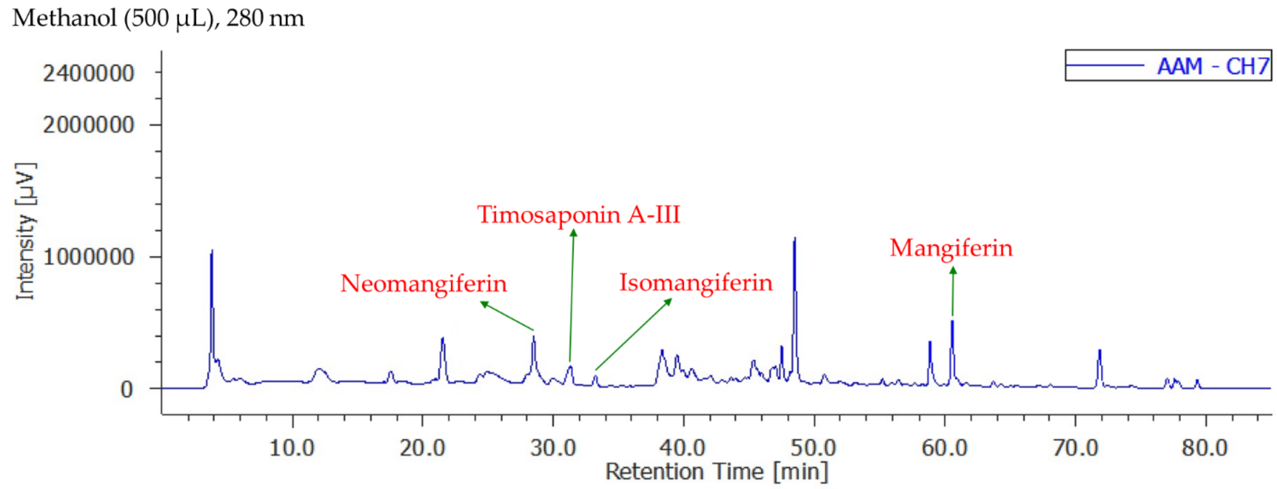

Figure S2. Reverse-phase HPLC chromatogram of methanol extract in AA.

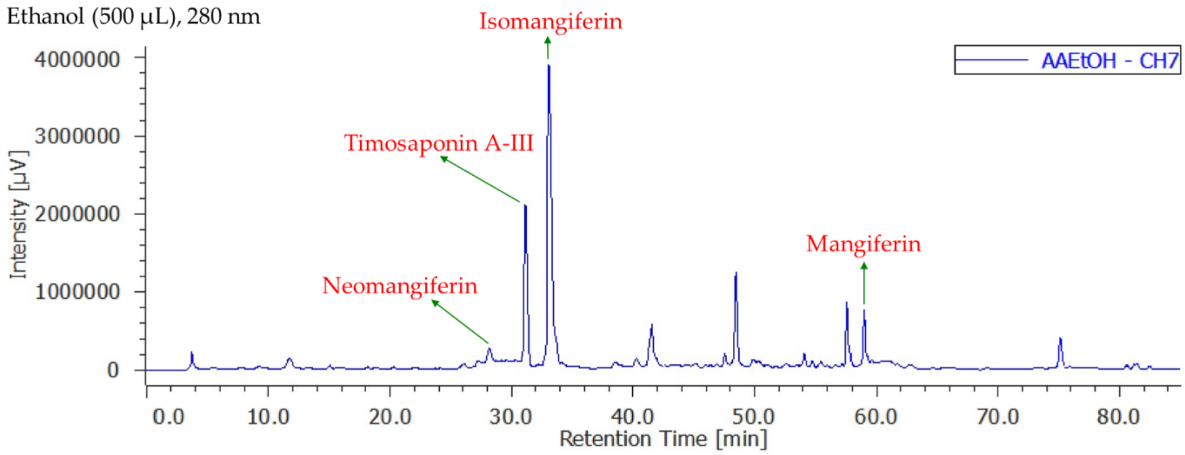

Figure S3. Reverse-phase HPLC chromatogram of ethanol extract in AA.

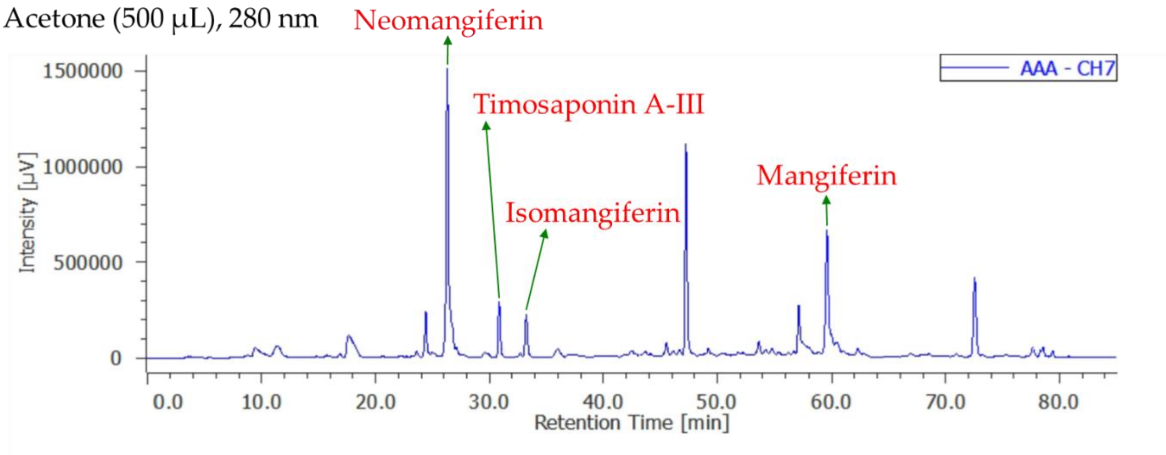

Figure S4. Reverse-phase HPLC chromatogram of acetone extract in AA.

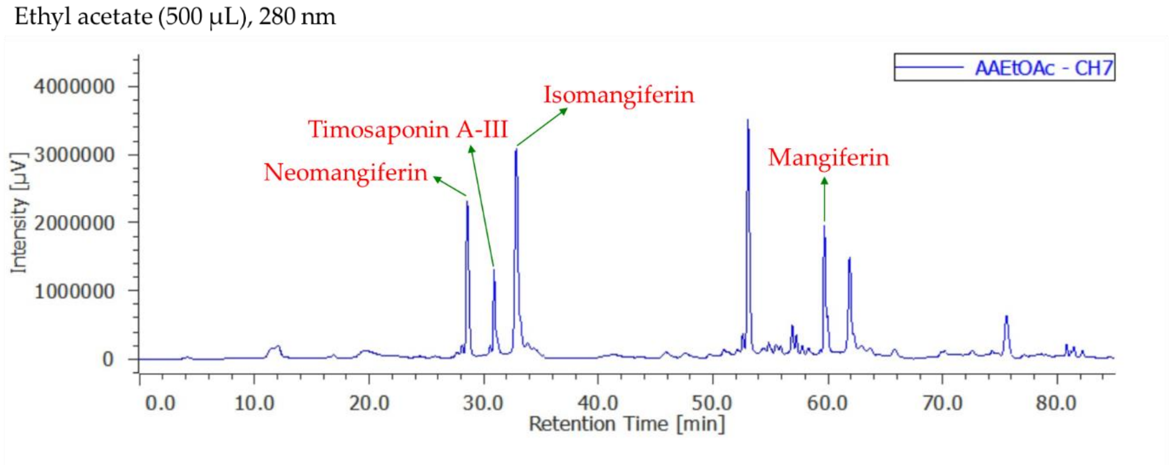

Figure S5. Reverse-phase HPLC chromatogram of ethyl acetate extract in AA.

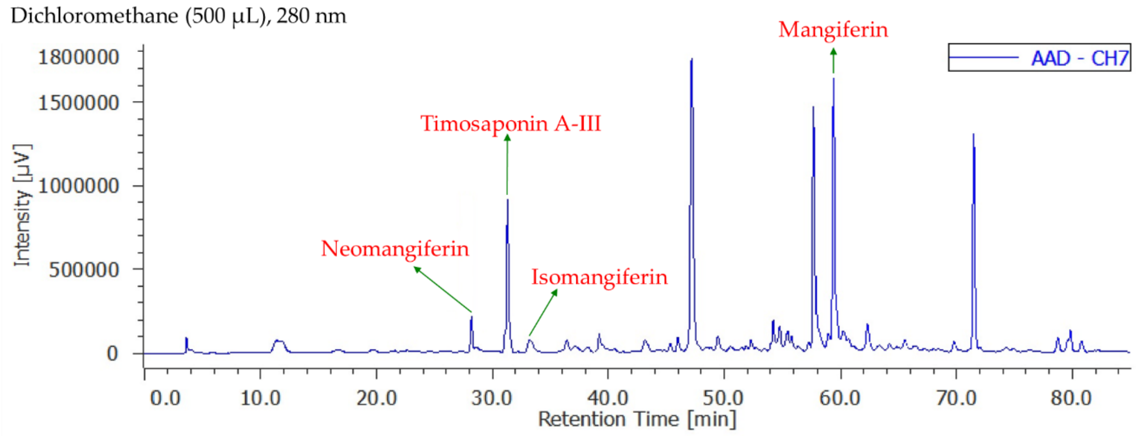

Figure S6. Reverse-phase HPLC chromatogram of dichloromethane extract in AA.

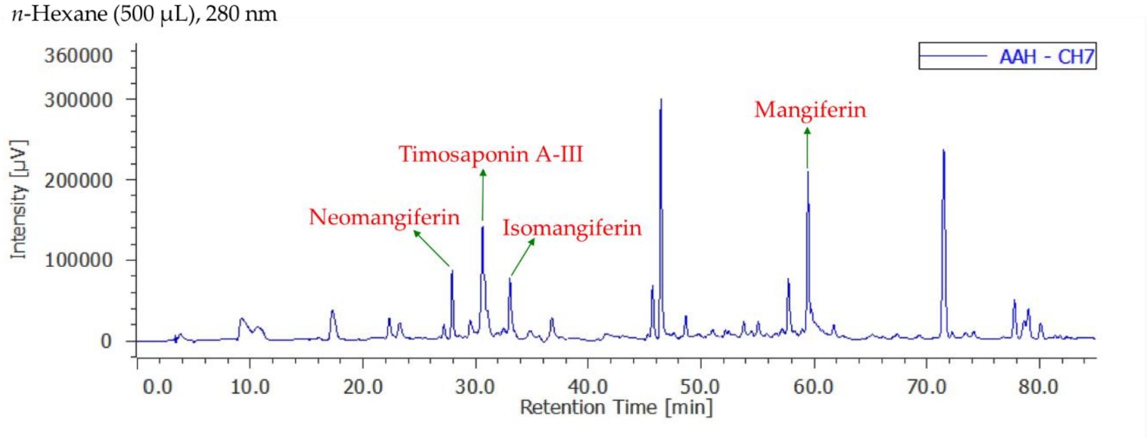

Figure S7. Reverse-phase HPLC chromatogram of *n*-hexane extract in AA.

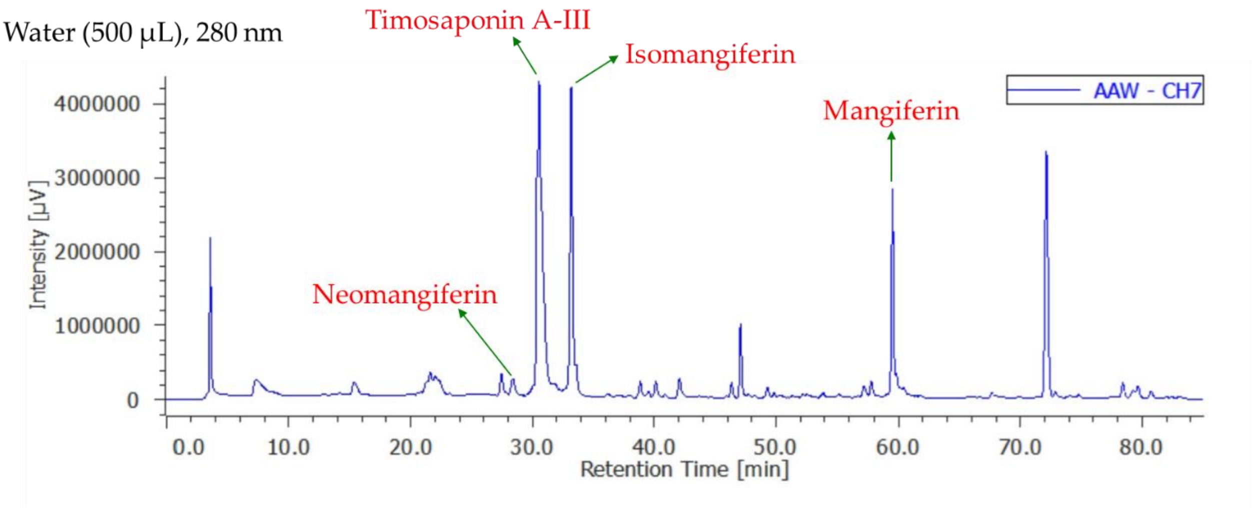

Figure S8. Reverse-phase HPLC chromatogram of water extract in AA.

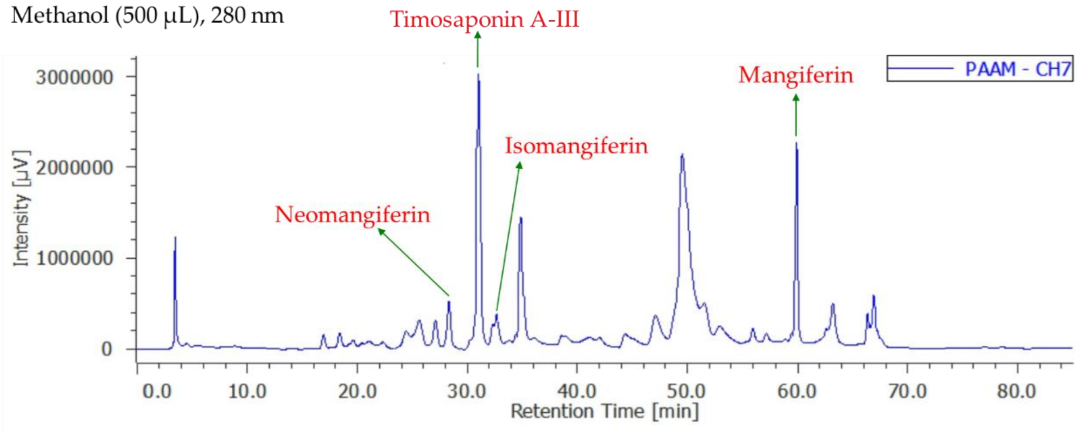

Figure S9. Reverse-phase HPLC chromatogram of methanol extract in salt-fried AA.

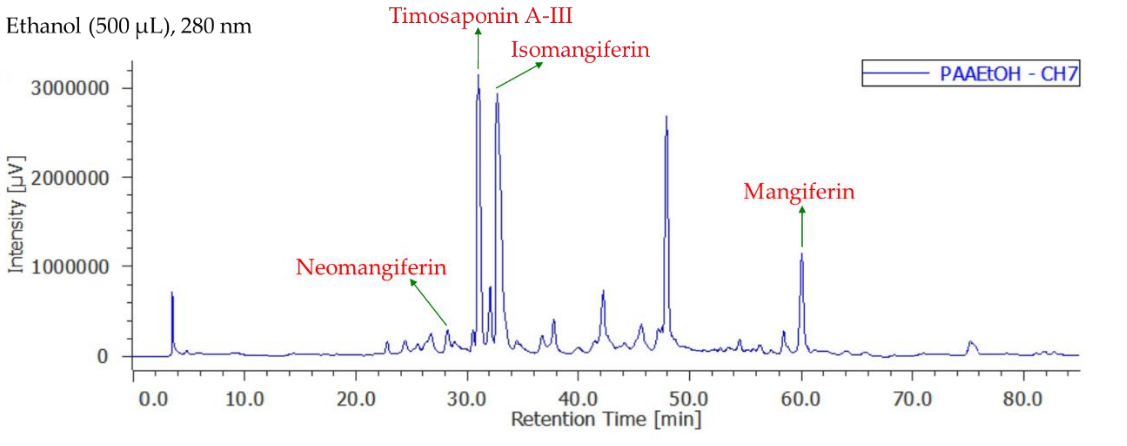

Figure S10. Reverse-phase HPLC chromatogram of ethanol extract in salt-fried AA.

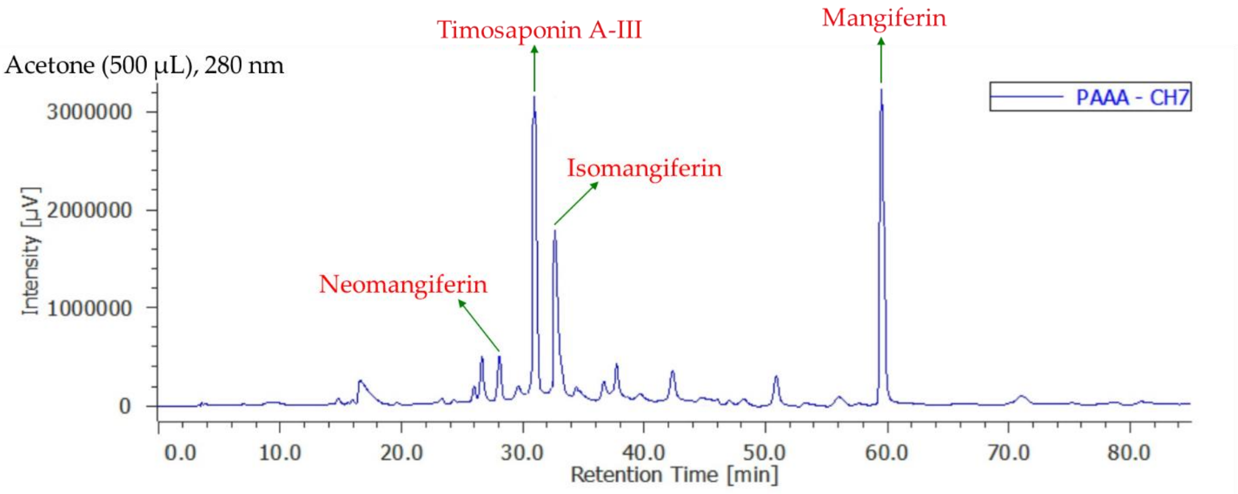

Figure S11. Reverse-phase HPLC chromatogram of acetone extract in salt-fried AA.

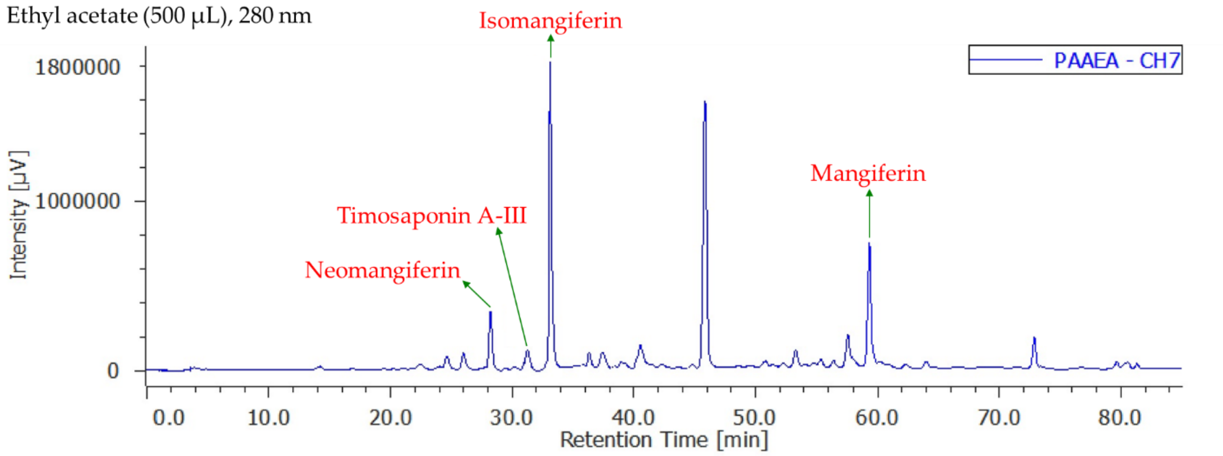

Figure S12. Reverse-phase HPLC chromatogram of ethyl acetate extract in salt-fried AA.

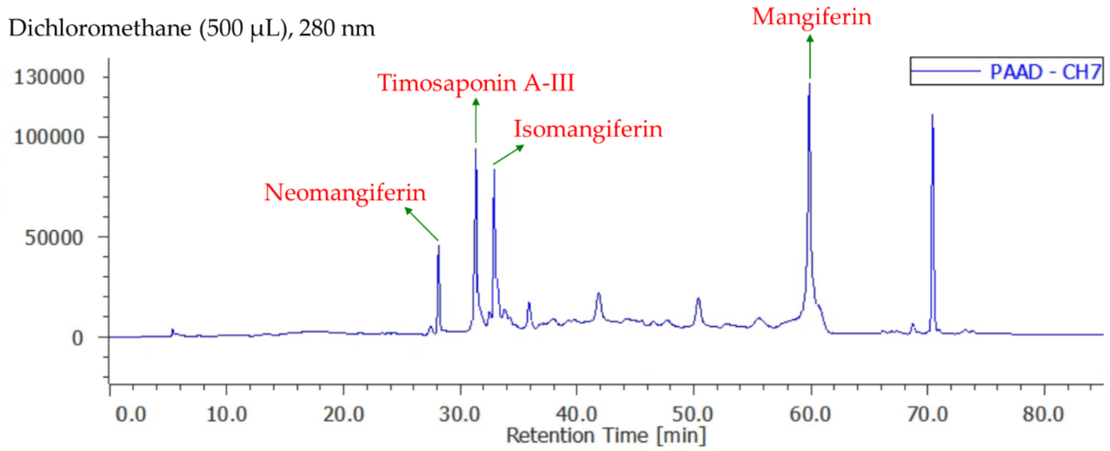

Figure S13. Reverse-phase HPLC chromatogram of dichloromethane extract in salt-fried AA.

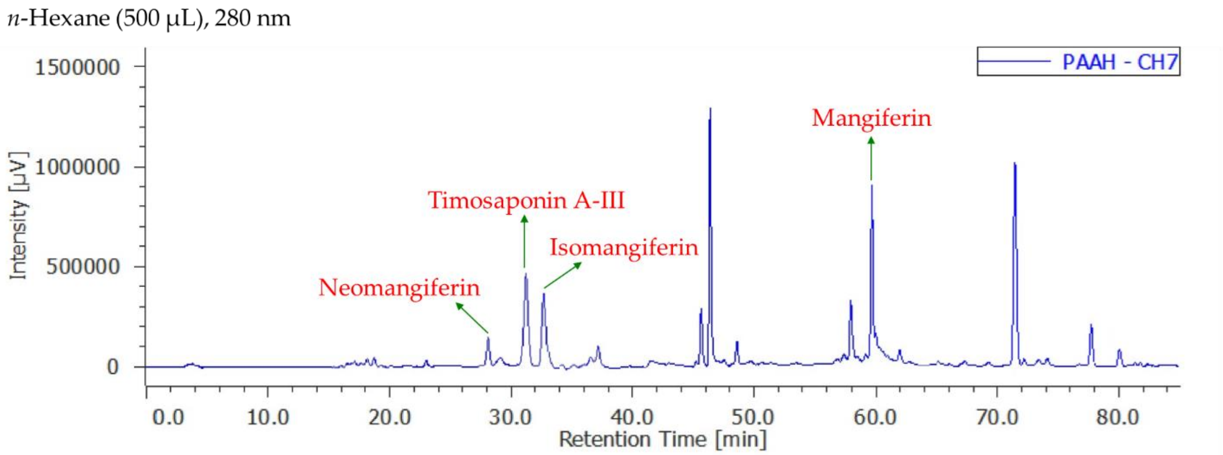

Figure S14. Reverse-phase HPLC chromatogram of *n*-hexane extract in salt-fried AA.

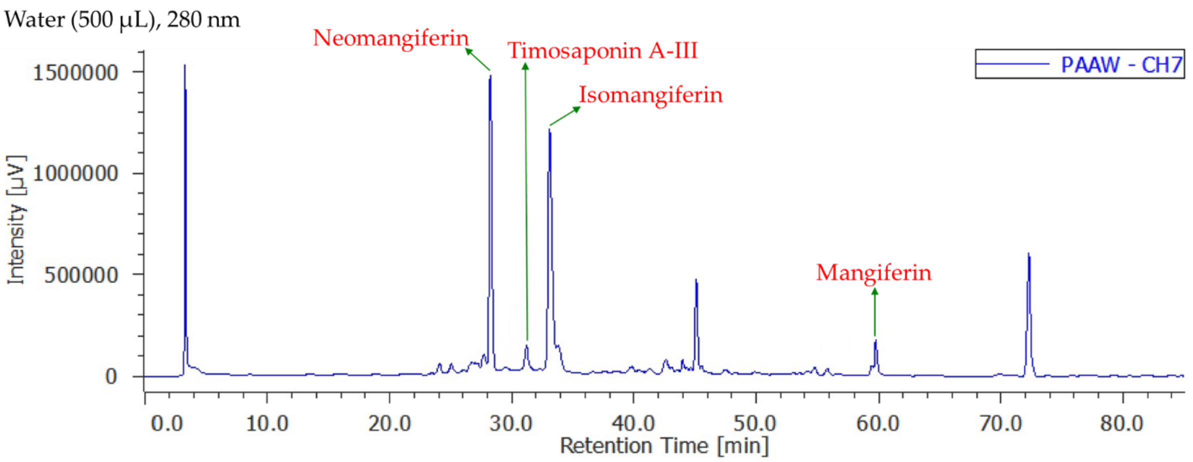

Figure S15. Reverse-phase HPLC chromatogram of water extract in salt-fried AA.
